# Digital health: a path to validation

**DOI:** 10.1038/s41746-019-0111-3

**Published:** 2019-05-13

**Authors:** Simon C. Mathews, Michael J. McShea, Casey L. Hanley, Alan Ravitz, Alain B. Labrique, Adam B. Cohen

**Affiliations:** 1 Armstrong Institute for Patient Safety and Quality, 750 E Pratt St, 15th Floor, Baltimore, MD 21202 USA; 2 Johns Hopkins Medicine, Department of Medicine, Division of Gastroenterology, 1800 Orleans St, Baltimore, MD 21287 USA; 3 Health Technologies, National Health Mission Area, The Johns Hopkins University Applied Physics Lab (APL), 11100 Johns Hopkins Road, Laurel, MD 20723 USA; 4 Johns Hopkins Bloomberg School of Public Health, JHU Global mHealth Initiative, 615N. Wolfe St, Baltimore, MD 21205 USA; 5 The Johns Hopkins Hospital, Department of Neurology, 1800 Orleans St, Baltimore, MD 21287 USA

**Keywords:** Health policy, Technology

## Abstract

Digital health solutions continue to grow in both number and capabilities. Despite these advances, the confidence of the various stakeholders — from patients and clinicians to payers, industry and regulators — in medicine remains quite low. As a result, there is a need for objective, transparent, and standards-based evaluation of digital health products that can bring greater clarity to the digital health marketplace. We believe an approach that is guided by end-user requirements and formal assessment across technical, clinical, usability, and cost domains is one possible solution. For digital health solutions to have greater impact, quality and value must be easier to distinguish. To that end, we review the existing landscape and gaps, highlight the evolving responses and approaches, and detail one pragmatic framework that addresses the current limitations in the marketplace with a path toward implementation.

## Introduction

The concept of digital health continues to evolve. Clinicians and patients should ask: Which solutions are substantiated and which, despite marketing claims, are not? First introduced in 2000 by Seth Frank,^1^ digital health largely encompassed internet-focused applications and media to improve medical content, commerce, and connectivity. The term digital health has expanded to encompass a much broader set of scientific concepts and technologies, including genomics, artificial intelligence, analytics, wearables, mobile applications, and telemedicine.^2^ In addition, digital health technologies are being applied much more broadly in medicine to include diagnosis, treatment, clinical decision support, care management, and care delivery. In 2018, the World Health Organization issued a detailed taxonomy of Digital Health, articulating dozens of facets of this expanding space.^3^

Investment in the digital health sector is enormous, with nearly $6B in funding in 2017, increased from $4.4B in 2016.^4^ For mobile health applications alone, there exist more than 3,00,000 health apps with more than 200 health apps added daily.^5^ This highlights the increasingly voluminous and cluttered landscape all healthcare stakeholders—patients, providers, payers, industry, and regulators—must navigate. Their challenge is finding solutions that provide real value.

Currently, no reliable mechanism exists to identify validated digital health solutions. Payers, too, cannot easily identify quality in this crowded field. Regulatory guidance and oversight are limited, with enforcement restricted to companies that make claims out of proportion to the evidence or where application failures might lead to risks to patient safety.^6^

Oversight frameworks of digital health have been proposed, which mainly focus on patient safety.^7^ Healthcare needs a robust and transparent validation process for digital health products. All healthcare stakeholders would benefit from a more standardized, objective, rigorous, and transparent process for validation. Specifically, the validation domains would be technical validation (e.g., how accurately does the solution measure what it claims?), clinical validation (e.g., does the solution have any support for improving condition-specific outcomes?), and system validation (e.g., does the solution integrate into patients’ lives, provider workflows, and healthcare systems). A proposed pathway is outlined. A forthcoming pilot study (and publication of a detailed corresponding framework) will contain finer details of the proposed pathway.

## Existing landscape

The rapid advancement and promotion of digital health technologies has produced a unique landscape, characterized by an industry able to rapidly iterate technology, often at the expense of the traditional medical product design, safety testing, and clinical efficacy trials. Prior to the digital and mobile age of health technology, technology development speed was set by manufacturing, distribution, and regulatory considerations, which inherently demanded a slower development cycle. Digital health technologies, however, generally lack these constraints. This enables rapid creation, iteration, and distribution, which incentivize developers to minimize the time to develop design requirements. The minimum necessary verification and validation activities are pursued to ensure correct product fabrication to meet the intended use and end-user needs. Although some digital health products have been rigorously studied to determine clinical effectiveness,^8,9^ such evaluation is not widespread. This can lead to discrepant and misleading claims, as well as dubious quality.^10^ For example, a recent evaluation of 280 diabetes mobile applications found only five associated with clinically meaningful improvement and none were of high methodological quality.^11^ This risk is further compounded by a dearth of well-established value and impact-based business models for digital health, which also leads to faster-paced experimentation. The “fail fast, fail often” mantra espoused by technology startups is frustrated by the confusing regulatory landscape of healthcare. This cultural clash is further exacerbated by the cautious, stepwise, and time-consuming process of healthcare innovation that is grounded in the risk-averse clinical principle of “first, do no harm”.^12^

**Fig. 1:**
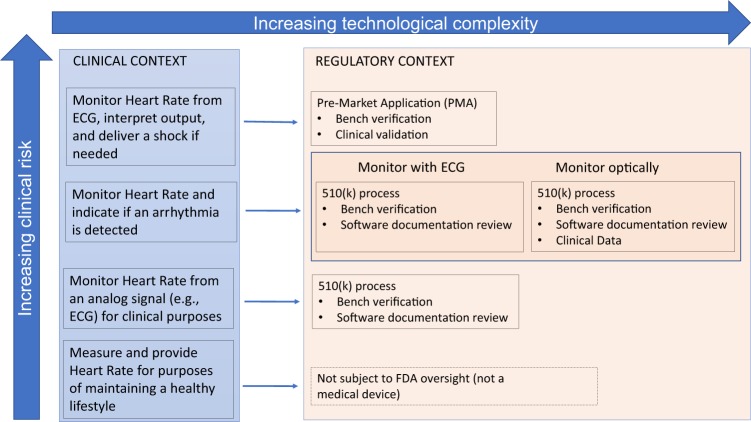
Regulatory–Clinical–Technology risk paradigm. Examples of increasingly complex clinical applications of technology and their corresponding regulatory contexts are presented in this figure

The use of health technology is also expanding into areas that are ambiguous from a regulatory perspective. For example, the 21st Century Cures act amended the Federal Food, Drug, and Cosmetic Act to remove certain clinical decision support functionalities from the definition of “medical device”, thus removing, or at least changing, the regulatory oversight responsibilities of these technologies by the Food and Drug Administration (FDA).^13^ In response, the FDA started pilot projects and published a number of guidance documents to elucidate their position on these topics.^14^ A representative illustration of the varying regulatory considerations based on clinical risk and technical complexity for digital technologies is shown in Fig. 1. Other Federal Agencies also have a role in regulation of digital health technologies. The Federal Trade Commission (FTC) prohibits deceptive or unfair acts or practices, including false or misleading claims about the safety or performance of digital health technologies. To provide clarity about the regulatory environment for a mobile app developer, the FTC, FDA, Health and Human Services (HHS) Office of the National Coordinator for Health Information technology (ONC), and Office for Civil Rights (OCR) have developed a tool for developers to use to understand what federal laws are applicable to their technology.^15^

While there remains significant regulatory ambiguity, these efforts represent initial efforts for clarity. There is not yet, however, commensurate attention paid to ensuring products meet target users’ needs. Current indications point to a future where only a fraction of digital health technologies are subject to regulatory review prior to market entry. Many other sophisticated, unproven solutions will continue to proliferate, frustrating end-users looking for a way to improve their well-being or disease self-management. Thus, the onus is shifted to the clinician and patient to identify effective and useful digital health technologies, bearing the risk of ineffective, or even potentially harmful, solutions. The need for new accessible tools to assist in informed decision-making is clear for all domains of the digital health spectrum illustrated in Fig. 1. For high-risk clinical scenarios or complex technologies, the necessity for such a tool is due to inherent concern posed by the technology, and would be useful to augment the FDA’s clearance or approval process. For lower-risk technologies where there is a trend toward lower regulatory oversight, new tools may provide the only independent insight into the performance of a digital health technology for consideration by the clinician and patient end-users.

## Evolving approaches

Initiatives to curate or certify digital health applications trace their roots back to early 2010, some even offering paid accreditation to help users separate “snake oil” from legitimate offerings.^16^ Several of these activities failed, rapidly overwhelmed by the Herculean task before them.^17^ Today, promising developments are underway to help better differentiate digital health products. A summary of existing representative resources and approaches are detailed in Table 1. Within industry, the Personal Connected Health Alliance provides design guidance based on the Continua Alliance standards and design model.^18^ This focuses on personal connected devices, such as weight scales, blood pressure monitors, and glucose meters. The newly formed Xcertia group is creating broad criteria and guidelines specifically for health mobile app curation, but leaves the task of evaluation and validation based on the guidelines to industry participants.^19^ This approach is in contrast to a notable predecessor of Xcertia, Happtique, a venture-backed company spinoff created by the Greater New York Hospital Association in 2010, which failed to commercialize health app certification. The Xcertia guidelines presently do not include clinical outcomes validation, though others such as NODE. Health and the Digital Therapeutic Alliance are attempting to fill this gap.

**Table 1:** Existing Resources

Existing resource/framework	History	Organizational membership	Focus	Reference	Areas scored	Methodology provided?	Ranking Criteria Defined?	Scoring transparent?	Standards-based assessment?
NHS	Apps Library launched in 2017 with 46 Apps	Open to all app developers to submit apps	Government sponsored validation effort using commercial company Our Mobile Health	https://digital.nhs.uk/services/nhs-apps-library https://developer.nhs.uk/digital-tools/daq/	Clincial effecdtiveness, regulatory approval, clincal safety, prvacy & confidentiality, security, usability & accessibility, interoperability, technical stability, change management	Yes	Apps are either NHS Approved, NHS under test; presumabily pass/fail	Somewhat	Yes
PCHA	Formed in 2014 as a spinoff of HIMSS, and merger with Continua Alliance	240 companies, including health providers, payers, pharma, medical device vendors	Connected devices and mobile platform interoperability and data standards; expanding into FHIR, direct ot cloud, cyber security	www.pchalliance.org	Health device interface, service interface, and healthcare information system interface	Yes	No ranking, certification only	Yes	Yes
Xcertia	Founded in 2016 by AHA, AMA, HIMSS, and DHX Group; Strategic alliance with Consumer Technology Association	Approximately 40 healthcare industry member companies, and liaison relationships with ATA, CTA, IEEE, EHNAC, PCHA.	Broad criteria for mobile app curation. Work groups created for security, privacy, content (evidence based), operability, and advocacy	http://xcertia.org/	Operability, content, security, and privacy	No; planned release in 2019	No	No	Yes, where standards exist.
RankedHealth	Run by the Hacking Medicine Insititute, a non profit spun off MITs Hacking Medicine program	N/A; Crowd Sourced medical and technology professionals perform peer reviews	Apps related to managing and monitoring chronic conditions and issues affecting broader populations, including mental health, heart disease, diabetes, obesity, sleep quality, fitness, medication adhernce, sumpton tracking, emergency care, pregnancy, and reproductive health	www.rankedhealth.com	Effectiveness: clincial relevance, credibility, evidence-based Functionality: features, data sharing, integration with other apps, HealthKit, or EMR Usability: user interface, user experience, ease of use, look and feeel	Peer review	No	No	No
NODE.Health	The Network for Digital Evidence in Health was founded in 2016	20 member health systems from across the health ecosystem; Health systems, Trade Organizations, corporations, accelerators, start-ups, payors	Vision of creating Evidence Based Medicine (EBM) for digital health solutions, ending “death by pilot”	www.nodehealth.org/	Clincial efficacy, and Usability (UX), as a necessary predicate to positive outcomes	Developed per solution as part of study design	Expected to be developed over time using data from multiple studies	Future vision	Yes, where standards exist.
Wellocracy	Sponsored by Partners Connected Health, founded by Brigham and Women’s Hospital and Massachusetts General Hospital, teaching affiliates of Harvard Medical School	Launched by Partners Center for Connected Health in 2013 to promote “Self-Health” consumer adoption of wellness apps.	Consumer fitness apps, nutrition, sleep, food and calorie, healthy habit and heart health apps and devics.	http://www.wellocracy.com/	Heuristic reviews of apps in each category, including consumer reports like relative comparisons. Main areas are Fun Features, “Which it Had”, comaptibility (device, iOS vs. Android), and consumer erviews.	Qualitative only	Yes; heuristic review by experts at Partners	No	No
Digital Therapeutics Alliance	Founded in October 2017 by Akili Interactive, Propeller Health, Voluntis, and Welldoc.	19 companies including primarily Pharma and pure play digital health solutions. 10 formal strategic advisors from industry and academia.	Digital health solutions that prevent, diagnose, or treat a medical disease or disorder or optimized medication. Predominantly in the regulated space.	https://www.dtxalliance.org/	Not a scoring mechamism but a framework with principles to be adapted, including product development practices, clinical validation, security and privacy, and promoting appropriate regulatory oversight of market claims and risks.	Framework of Principles only	No	No	No

This table provides representative examples and details of existing resources that aim to address various aspects of digital health evaluation

The United Kingdom’s National Health Service (NHS) currently hosts a Beta site Digital Apps Library, in which it lists “NHS approved” 70 + medical apps across an array of medical conditions, including cancer, chronic obstructive pulmonary disease, dementia, diabetes, mental health, and pregnancy.^20^ These aim to reflect high-quality, safe, effective, accessible, and usable apps for which published medical evidence supports their use. Other listed apps are “Being Tested in the NHS,” which reflect preliminary approval and current research within the NHS to determine clinical effectiveness. The NHS specifically solicits from industry app development for particular areas of health, including maternity, social care, chronic conditions, cancer, and mental health. Broad criteria for approval, defined by NHS Digital and National Institute for Health and Care Excellence, pertain to clinical effectiveness, regulatory approval, clinical safety, privacy & confidentiality, security, usability & accessibility, interoperability, technical stability, and change management. A host of additional app curation sites also exist,^21,22^ with a varying range of analytic methods compared with the NHS.

These initiatives are relatively new with overlapping standards. They will likely advance the field, but may not provide sufficiently clear and robust direction for patients and providers on the most effective products that meet specific requirements to best integrate into a particular healthcare context. In addition, there is a lack of objective, comprehensive, transparent, and standards-based evaluation, which also limits confidence and practical application of existing approaches.^23^ A Digital Health Scorecard—a multi-stakeholder approach that objectively and rigorously evaluates solutions—is needed to provide the healthcare marketplace with greater insight, clarity, and guidance.

Outside digital health, there are analogous consumer product evaluation organizations, such as UL (formerly Underwriters Laboratory)^24^ and Consumer Reports,^25^ which provide potentially useful models for digital health. UL focuses on safety-related topics by certifying product compliance with international standards. UL does not, however, ensure a product meets end-user needs. Although safety is one end-user need, needs extend beyond safety, including portability, interoperability, and usability. Consumer Reports, on the other hand, represents the opinions of objective reviewers regarding the ability of a product to serve a particular function, which is end-user focused—is the product right for the job? Together, UL and Consumer Reports evaluations steer product developers toward the development of solutions that best serve end users. There are two important differences between UL and Consumer Reports. First, the standards UL certifies are well-known to the product developers. Second, UL conducts pre-market certification testing. In contrast, the requirements Consumer Reports uses as the basis for its evaluations are elicited post market and are not well documented or disclosed to the developer pre-market—the developer must anticipate end-user needs and requirements and therefore hope to “get it right.” Our proposed Digital Health Scorecard is based upon a hybrid approach that proactively defines requirements and standards for digital health products; transparently discloses them; and objectively evaluates and reports to industry and the public.

## Need for requirements-driven approach

**Fig. 2:**
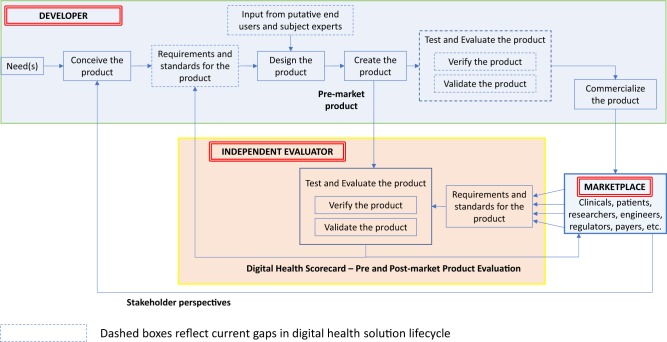
Traditional Product Lifecycle with Proposed Digital Health Scorecard Added. A representative depiction of the steps within a traditional product development lifecycle is presented in the top half of the figure. The role of an independent evaluator and its relationship with the broader marketplace and product lifecycle is also presented

In aviation or motor vehicle manufacturing, there is a straightforward path from a perceived market need to product commercialization (Fig. 2, top box). The product lifecycle begins with establishing requirements. Here, teams of stakeholders define the features, functionality, and performance needs for the product—“the product shall do X” and “the product shall not do Y.” (Requirements may contain great detail on what the system should not do.)

An aviation requirement, for example, might state “the airplane shall provide seating for no more than six people.” Further context for that requirement might include nominal or maximum size and weight of those adults. Furthermore, requirements could dictate standards, such as electrical or physical safety standards. Importantly, these requirements and standards are thoroughly documented and may even lead to initial prototypes, which may be virtual simulations or physical models of the eventual end product to aid in refining the design ensuring it meets the specified requirements. The crucial “Test and Evaluate” step focuses on verification and validation. The former term determines whether the product was designed and developed in accordance with the upfront requirements (e.g., does the plane accommodate six passengers?), while the latter determines whether the product meets the end-user’s requirements (e.g., should it have been designed to carry more people?).

Presently, much of the digital health industry lacks this rigor, including several steps along the traditional product development cycle (Fig. 2, dashed boxes). The current digital health product lifecycle often focuses on high-level requirements, if at all, which limits what can be verified or validated. These fundamental elements of the product development process remain essential even in today’s world of agile development, and continuous cloud-based product deployments. We believe a Digital Health Scorecard will promote requirements-driven development to the benefit of all stakeholders.

Specifically, in our proposed lifecycle (Fig. 2), an “independent evaluation” phase provides a role, both during development and post-market entry. In this approach, an objective independent entity (or authorized entities) undertake the evaluation of digital health pre-market products emerging from developers. The independent evaluation would address both verification and validation and would be based upon a set of well-defined (i.e., measurable, concise, unambiguous) requirements. Developers can use feedback from the Digital Health Scorecard to refine existing products or create new ones. The broader marketplace could use the Scorecard to make informed decisions regarding which products are most applicable for the intended use and which products perform best.

## Components of a digital health scorecard

**Fig. 3:**
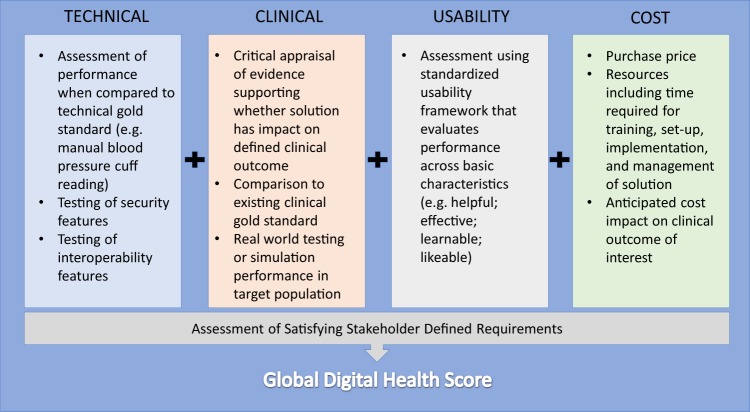
Components of Digital Health Scorecard. The four domains of a digital health scorecard with example considerations are detailed in this figure. Their relationship to an assessment of stakeholder requirements is also presented

The development of requirements will vary across types of digital health solutions based on functionality (diagnostics, monitoring, care coordination, etc.), which can also be modeled from other industry approaches. It is critical to incorporate the preferences of the clinicians and patients impacted by the digital health solution into the requirement development process. Once requirements are established, the proposed framework that could form the basis for evaluation includes the following domains: technical, clinical, usability, and cost (Fig. 3).

### Technical

Technical validation is the most traditional type of evaluation in product testing. Does the solution actually perform to its self-proclaimed functionality with accuracy and precision? For example, how accurately and reliably does a wearable measure heart rate compared with a gold standard? Other elements of technical validation could also include security and interoperability assessments. Applications that claim to perform the functions of established medical devices, such as those reflecting biological processes like heart rate, blood pressure, or respiratory rate, should be able to demonstrate equivalence according to rigorous standards set for other non-smartphone based novel devices.^26^ System failure and redundancy must also be considered—what happens if a digital health system degrades over time as phone camera lenses become scratched or occluded? Is there a need for back-end monitoring of user engagement or daily calibration to ensure appropriate system performance? Do certain processors or sensors fail to meet stringent minimum standards required for reliable system outputs?

A system architecture, the sum of structure, behavior, and components of a technology, has unique considerations in digital health. Developers must consider the privacy and security requirements of handling patient data that may be confidential and even linked to larger electronic health record systems. Robust, enterprise architecture standards exist to guide developers on issues, such as the levels of encryption and user authentication necessary to safeguard patient information. This requirement may vary with the degree of inherent confidentiality of the health condition in focus— ranging from low, when dealing with step-counters or accelerometers—to high if managing a socially stigmatized disease or storing test results.

### Clinical

Clinical validation to demonstrate efficacy is generally well understood and considered vitally necessary in the context of traditional clinical or translational research. However, analogous studies for digital health products is uncommon.^27^ This gap may be due in part to the lack of clinical subject-matter experts engaged in the digital health product development.^28^ Regardless of what part of the continuum of care (prevention, detection, and management) the product addresses, a validation study must compare it to relevant clinical gold standards. Particularly for studies aiming to demonstrate the clinical impact of the a product, these may take the form of accepted care quality metrics, such as measures of clinical outcomes (e.g., presence of disease or complications, clinical functional scores) and process (e.g., laboratory values, adherence to treatment guidelines).

A digital health product that aims to prevent diabetes mellitus would be measured by standard clinical quality measures, such as clinically validated disease diagnostic criteria,^29^ glycemic control, or diabetes-specific complications like stroke and retinal disease.^30^ This is important not only to ensure that content is based on current state-of-the-art evidence and guidelines but even to assure users that simple digital interventions, such as behavior-modification applications leverage and incorporate decades of proven, efficacious strategies.

An integral validation step is standardized and critical appraisal of any existing evidence (e.g., an approximation of the rigorous GRADE system^31^) to support any clinical claims made by the product or solution. Existing digital health review resources may provide subjective assessments of the quality of clinical evidence by technical experts, but generally do not provide systematic and objective analysis.

More advanced validation efforts would require external testing within a simulated or actual trial settings to determine if results can be duplicated. Furthermore, such testing would determine how the product performs across the relevant provider systems, clinical settings, and integrated technologies, in which it is likely to be deployed. For example, the data that flows from an app that monitors cardiac function should actually assess the intended cardiac measure or function, assess it in the targeted population (e.g., congestive heart failure), and be accessible to the clinics and hospitals relevant to the target patient population. The data must flow across emergency, intensive care, and ambulatory settings to electronic health records, provider alert systems, and ambulances of these hospital systems. Without meeting such system requirements, deployment of such an app, even if shown to be clinically effective in a research setting, may have no meaning in the real healthcare system for patients.

An added consideration for “prescription-strength” digital health applications may involve post-market surveillance (PMS), as it is required for most drugs and medical devices.^32^ Often, pre-release clinical studies are not powered to detect rare, but serious, potential device failures which may cause unintended harm to users. Currently, the FDA monitors reports of device and drug adverse events and may require PMS to clarify possible adverse events associated with a device—or verify that efficacy of a device persists in the “real world”, especially if clinical tests used a surrogate outcome for the purposes of expediency.^33^

### Usability

Formal usability assessment of traditional healthcare goods and services (e.g., pharmaceuticals, procedures, and treatment plans) is frequently embedded within the regulatory and patient experience pathways that lead to their development. Similarly, usability testing in the setting of medical devices is common (from a clinician/operator perspective) due to regulatory requirements, although generally with a safety focus.

When considering digital technology, there is no assurance or protection that the technology will align with user needs or preferences. Usability is arguably among the most important considerations with patient-oriented mobile- and digital-based solutions. These technologies are frequently literally in the hands of patients and consequently demand a more patient-centered approach to usability. Existing digital health qualitative reviews address only some aspects of usability. All stakeholders would benefit from a standardized approach, unlike the status quo, which is often ad hoc, qualitative, or dependent on the volume of reviews.^34^

Digital health apps must be easy to use for their intended purpose, require minimal effort to complete tasks, have minimal data entry burden, and allow the user to control preferences when appropriate (e.g., notifications). Since systems can be designed for users with different requirements (e.g., impaired vision, motor deficits, cognitive dysfunction), design considerations must reflect the target user-audience.

The World Wide Web Consortium has summarized a user-centered design process (UCD),^35^ which takes into account multiple frameworks and mirror some elements of the International Organization for Standardization’s multi-part standard, ISO 9421.^36^ The key objectives outlined in this standard are applicable to the evaluation of digital health applications: the solution should be useful in helping users achieve their goals, effective (i.e., producing results with minimal user error), learnable (i.e., easy and intuitive to use), and likeable (i.e., enjoyable to use). These considerations, not surprisingly, play an important role in patient engagement—an often neglected, yet essential aspect of digital health; having a unused medical device is tantamount to not having one at all.

At a minimum, a best-practice evaluation framework should be considered,^37,38^ but these frameworks establish a lowest common denominator, and do not necessarily incorporate the principles of user centered design into the development process. There are multiple efforts underway to codify standards for design principles specifically to digital health solutions, most notably Xcertia and Node.Health. Some criteria are easier to objectively specify than others. For example, criteria such as number of steps required to complete essential tasks, consistency of navigation, visual legibility, and use of recognizable iconography can be objectively developed. More work is needed to formalize subjective aspects of usability, such as utility and user delight and satisfaction. Usability viewed in this way, along with clinical relevance, creates the opportunity to impact patient engagement.

To maximize impact, digital health solutions will likely require clinician input as part of solution development, thereby accounting for UCD on at least two fronts. Just as early electronic health record implementations increased clinician burden by not adequately considering clinician workflow,^39^ digital health solutions designers need must pay attention to ease of accomplishing the expected tasks.

### Cost

At face value, cost—defined as the price a consumer must pay to gain access—may be an inadequate differentiator of digital health solutions—many are free or low priced, particularly in the mobile app arena. When integrated in a composite assessment, however, true cost may provide greater discrimination of overall value. Here, cost estimation becomes more complex by incorporating broader considerations, such as costs of the technology lifecycle and those to integrate technology into the clinical workflow.

Furthermore, the long-term cost implications from outcomes improvements are also difficult to calculate but should be taken into consideration, leveraging metrics from pharmaceutical and device industries. While determination and attribution of financial benefit derived from mobile health apps is challenging,^40^ real value may be derived from increased personal health engagement, improved patient–clinician engagement, or patient and clinician satisfaction. New ways of quantifying and measuring these types of attributes will provide a more comprehensive picture overall cost-benefit.

### Global score

In the consumer financial industry, the FICO score represents a global score as an amalgamation of credit information to approximate borrower quality and lending risk.^41^ It contains five subdomains: payment history, current level of indebtedness, types of credit used, length of credit history, and new credit accounts. Correspondingly, by aggregating the individual domain assessments from the Digital Health Scorecard and contextualizing the degree to which a product satisfies end-user requirements, a composite Global Digital Health Score can be created. As a result, consumers and other users would be provided a high-level synthesis of quality and risk for digital health products.

This aggregate score allows gross initial selection of digital solutions. Individual scores allow finer discrimination of particular products. Such scores also allow digital health companies to identify where improvements are needed and inform stakeholders on what gaps will exist when the products are deployed. The scores could become benchmarks and establish thresholds for particular types of digital solutions. These scores would also need to highlight and prioritize how well the product ultimately met the end-user requirements.

### Challenges

The successful Digital Health Scorecard allows better discrimination of digital health products, but just as important, pushes digital health companies to build impactful products that work for real patients, providers, and healthcare systems. Three main types of barriers (conceptual, financial, and operational) could hinder broad execution of this approach.

### Conceptual challenges

The framework described is one possible approach to characterize the needs in digital health and may not fully reflect the needs of the current marketplace. Our framework is modeled from other industry approaches and needs we perceive for digital health. Given the multi-stakeholder nature of healthcare and varying stakeholder incentives, the best approach to impactful, useful, and integrated digital health products may differ than the one described here. For example, although our approach aims to maximize clinical impact on patients, a payer may be more interested in efficient resource utilization. A provider system may seek products that boost high-reimbursement activities, such as attracting surgical patients or increasing advanced, high-cost imaging utilization. Thus, our proposal may not be practical for or applicable to every permutation within the current fragmented healthcare landscape, in which different stakeholders have different incentives. We believe our approach, however, would promote a requirements-driven, impact-focused digital health landscape. Given the nascent, shifting nature of digital health, the most realistic initial approach to a Digital Health Scorecard implementation is to create a requirement set broad enough to encapsulate concepts important to all products, but not inclusive of so much detail that the requirements are not realistic or relevant.

### Financial challenges

The Digital Health Scorecard concept requires a financially viable business model for implementation and sustainability. Sustainability could be realized if companies saw value in, and thus purchased Digital Health Scorecard reports or accreditation ratings. If patients, providers, or payers also saw value in or demanded these reports and ratings, companies may feel or be required to purchase them. It is conceivable that payers and even hospital, provider, and medical specialty associations would fund the creation of such reports and ratings, particularly as they become more financially incentivized to adopt digital health products that improve outcomes.

### Organizational and operational challenges

From an organizational perspective, unlike aviation and the construction of airplanes, digital health products have no single owner of the requirements. The Federal Aviation Administration and aviation companies, however, can set specific requirements that guide the development of airplanes. In digital health, neither patients, physicians, hospitals, payers, nor governmental regulatory bodies create or abide by standard requirement sets for digital health products. Also, unlike conventional aircraft design, many digital health products and accessories are so new that the desired or optimal requirements may be unknown. This further complicates which stakeholder should take primary ownership of driving requirements. No centralized body exists to serve as a clearinghouse for digital health application feedback or failure reporting. Like digital health products, however, novel aviation products such as drones, face unstandardized requirements without a clear curator of these requirements. For all new product types, relevant stakeholders should develop broad requirements categories relevant to the product and a corresponding scorecard approach that enables a best-practice product development and robust product assessment.

From an operational perspective, the tremendous volume and growth of products presently precludes rigorous evaluations for all products. Thus, a Digital Health Scorecard would likely fail in the current landscape if applied universally. Initially, a pragmatic approach could be applied to select products, such as those relevant to high burden or high-cost conditions, already popular with patients or providers, or with peer-reviewed studies to demonstrate validation, efficacy, or both. In addition, self-initiated industry evaluation may overcome the practical barrier of creating a fully capable Digital Health Scorecard organization that tests and evaluates products. Companies could be successful if a clear Digital Health Scorecard guided them with product development. The Scorecard itself could present an opportunity for a multi-stakeholder consultation to first generate and then periodically update, as refinements emerge with time and experience.

If independent outside assessment of digital health products is to be realized, scaling up assessment to meet the demand of many new products would require a large organization, certification network, and substantial resources. The latter could be accomplished by identifying or building a network of independent, objective organizations that could complete the different domain assessments, while following universal requirements from the Scorecard. Partners could vary by their role: traditional hardware/software testing firms to perform validation assessments, academic institutions to perform clinical studies, and auditing firms to assess cost.

### A way forward

The road to validating digital health will take resources, collaboration, and time. Even if successful, the first iteration of the Digital Health Scorecard will be different than latter versions as the healthcare environment evolves. In particular, a Scorecard approach will be most successful when all stakeholders partake in its construction and all stakeholders’ financial incentives are aligned to outcomes. The first Scorecard version, however, if transparent, rigorous, and pragmatic, would be an important step toward impact-driven digital health products that function in real healthcare settings. We are presently pursuing a small-scale pilot study implementing this approach in granular detail, the results of which will be published upon completion.

We believe there are two non-mutually exclusive initial approaches to the Digital Health Scorecard model. First, governmental regulatory bodies should partner with clinical stakeholders to create a standard set of requirements using the categorical concepts proposed here. The regulatory effort could be driven by one organization, such as the FDA’s Digital Health arm, or a collaboration between varied agencies like the FDA, FTC, the Centers for Medicare & Medicaid Services, and the ISO. This collaboration could produce certifications and ratings, but most importantly, requirements sets that promote the development of digital health products poised to be efficacious for the target patient population and function in a healthcare system with varied stakeholders. We do not expect one regulatory body to have the bandwidth, resources, or expertise to take this on alone.

Second, a provider healthcare or hospital system (or collection of systems) could lead to the development and adoption of a Digital Health Scorecard. Our preference is for a hybrid approach in which leading hospital systems partners with one or more of the aforementioned regulatory bodies, including the FDA, to lead a requirements-driven Scorecard approach. This system would not only lay out the requirements for digital health products that enter it, but only accept products that achieved high marks for each category of assessment. An existing healthcare system with enough patient volume could have ample market clout to influence the development of digital health products. Further, healthcare systems could collaborate to create an even more influential Scorecard model of care that drives requirements and product development across larger geographic regions and patient populations.

Although many large healthcare systems have sufficient market influence to drive this change, most are not nimble enough to do so. For example, many leading healthcare systems remain tied to electronic health record technologies, nearly universally disparaged by end-user practitioners and healthcare leaders.^42^ Furthermore, although many such systems desire to implement digital health and telemedicine care models, they largely represent a small fraction of clinical care.

The healthcare environment is evolving and converging with the entrance of non-traditional healthcare players, such as the CVS-Aetna partnership^43^ and the nascent Amazon–JP Morgan Chase–Berkshire Hathaway collaboration.^44^ Here, healthcare systems unconstrained by typical barriers to innovate are perhaps more realistic and promising champions of requirement-driven digital health.

As digital health companies have become more prolific and the number and diversity of digital health products has multiplied, the need for requirement-driven product realization and systematic validation has become increasingly important. Patients and providers will benefit from and demand the ability to discriminate clinically meaningful solutions. Payers and investors will need to identify high-value opportunities that ultimately guide reimbursement, investment decisions, and impact-focused care. Given the growing pull from non-industry stakeholders to demand impact-focused, interoperable digital health products, industry will also find utility in demonstrating product quality over product claims. We provide a framework to guide the evolution and successful delivery of validated digital health solutions.
